# A Histone Deacetylase Inhibitor Suppresses Epithelial-Mesenchymal Transition and Attenuates Chemoresistance in Biliary Tract Cancer

**DOI:** 10.1371/journal.pone.0145985

**Published:** 2016-01-04

**Authors:** Takuya Sakamoto, Shogo Kobayashi, Daisaku Yamada, Hiroaki Nagano, Akira Tomokuni, Yoshito Tomimaru, Takehiro Noda, Kunihito Gotoh, Tadafumi Asaoka, Hiroshi Wada, Koichi Kawamoto, Shigeru Marubashi, Hidetoshi Eguchi, Yuichiro Doki, Masaki Mori

**Affiliations:** 1 Department of Surgery, Graduate School of Medicine, Osaka University, Yamadaoka 2–2 (E2), Suita, Osaka 565–0871, Japan; 2 Department of Surgery, Osaka Medical Center for Cancer and Cardiovascular diseases, Nakamichi 1-3-3, Higashinari-ku, Osaka, Osaka 537–8511, Japan; University of Alabama at Birmingham, UNITED STATES

## Abstract

Epithelial-mesenchymal transition (EMT) is involved in the characteristics of malignancy, such as invasion, metastasis, and chemoresistance. In biliary tract cancer (BTC), EMT is induced by transforming growth factor-beta 1 (TGF-β1). The EMT is reversible; therefore, it is conceivable that it could be related to some epigenetic changes. We focused on histone deacetylase (HDAC) inhibitors as regulators of TGF-β1 signaling, and investigated their effect on EMT and chemoresistance. We employed four BTC cell lines (MzChA-1, gemcitabine-resistant MzChA-1, TFK-1, and gemcitabine-resistant TFK-1) and used vorinostat as the HDAC inhibitor. The relative mRNA expression of an epithelial marker (CDH1) and mesenchymal markers (CDH2, vimentin, SNAI1) were measured by qRT-PCR to evaluate factors associated with EMT. MTT (3-(4,5-Dimethylthiazol-2-yl)-2,5-diphenyltetrazolium bromide) assay was performed to evaluate the chemoresistance of each cell line. In addition, NOD/SCID mice were used to evaluate the effect of vorinostat *in vivo*. In the parent MzChA-1 and TFK-1 cell lines, TGF-β1 induced EMT and chemoresistance; while vorinostat inhibited the EMT and chemoresistance induced by TGF-β1. In gemcitabine-resistant cell lines that highly expressed TGF-β1, vorinostat inhibited EMT and attenuated chemoresistance. We showed that vorinostat inhibits nuclear translocation of SMAD4 which is a signaling factor of TGF-β1, and this is one of the mechanisms by which vorinostat regulates EMT. We also showed that vorinostat attenuates the binding affinity of SMAD4 to the CDH1-related transcription factors SNAI1, SNAI2, ZEB1, ZEB2, and TWIST. Furthermore, combination therapy with vorinostat and gemcitabine improved survival time in the mice xenografted with gemcitabine resistant MzChA-1 cells. In conclusion, vorinostat regulated TGF-β1-induced EMT and chemoresistance through inhibition of SMAD4 nuclear translocation.

## Introduction

Biliary tract cancer (BTC), which has an increasing incidence worldwide, has a poor prognosis because it is difficult to diagnose in the early stages. The 5-year survival rate is less than 30% [1–3]. At diagnosis, many patients have unresectable BTC due to local disease spread and/or metastases. Gemcitabine (GEM)-based chemotherapy is often used; however, the antitumor effect is insufficient, and the median survival time is limited to approximately 10 months [4,5]. To improve the prognosis of BTC patients, the molecular biology of the disease has been studied, including the inflammatory cytokines, epithelial-mesenchymal transition (EMT), DNA-repair system, cell signaling, and apoptosis [6–9]. We previously showed that EMT was observed at the invasion front and in regional lymph node metastases with inflammatory cytokine expression in BTC [10]. Several kinds of cytokines reportedly induce EMT, which is related to malignant processes such as invasion, metastasis, and chemoresistance (S1 Table) [11–15]. Transforming growth factor-beta 1 (TGF-β1) induces EMT and chemoresistance, and chemoresistant BTC cells produce IL-6 and TGF-β1 [10]; therefore, we considered that these cytokines could play an important role in promoting EMT in BTC.

Consecutive rounds of EMT and mesenchymal-epithelial transition (MET) occur during tumor development, suggesting that EMT could be reversible and associated with epigenetic changes [16]. We focused on histone deacetylase (HDAC) as a factor related to epigenetic changes. In our microarray data, the mRNA levels of class I HDACs (HDAC1, HDAC2, HDAC3, and HDAC8) were elevated in GEM-resistant BTC cells. Moreover, the HDAC activity was increased by exposure to TGF-β1. These findings suggest that HDACs could be associated with EMT, and inhibition of HDACs may suppress EMT through effects on TGF-β1 signaling. We also examined SMAD4, the key signaling factor of TGF-β1 for EMT, and found it was directly stimulated by TGF-β1 signaling, with translocation into the nucleus where it upregulates EMT-related transcription factors such as SNAI1, SNAI2, ZEB1, ZEB2, and TWIST [17–21]. Additionally, in normal hepatocytes and mesothelial cells, inhibition of HDACs attenuated the nuclear translocation of SMAD4 [22,23]. In this study, we hypothesized that HDAC inhibitors might regulate EMT through the TGF-β1 signaling pathway in BTC cells.

The aim of this study was to investigate the effect of the HDAC inhibitor vorinostat on the TGF-β1 signaling pathway and chemoresistance, which is related to EMT in BTC cells. First, we investigated the effect of vorinostat on TGF-β1-induced EMT. Then, we investigated the effect of vorinostat on chemoresistant BTC cells. Subsequently, we showed the influence of vorinostat on SMAD4 nuclear translocation in BTC cells. Finally, we evaluated the efficacy of vorinostat combined with GEM for BTC with using mice xenografted with MzChA-1 or GEM-resistant MzChA-1 cells. These results showed the effectiveness of a HDAC inhibitor in regulating EMT and chemoresistance *in vitro* and *in vivo*. We also identified one of the mechanisms by which a HDAC inhibitor suppresses EMT and attenuates chemoresistance.

## Materials and Methods

### Cell lines, cultures, and drugs

We used the human BTC cell lines MzChA-1 and TFK-1. The MzChA-1 cells were kindly provided by Prof. Gregory J. Gores of the Mayo Clinic, Rochester, Minnesota, United States of America (USA) [24–27]. The MzChA-1 cells were propagated in Dulbecco’s modified Eagle’s medium.

The TFK-1 cell line was obtained from the Riken BioResource Center in Japan. These cells were propagated in RPMI 1640 medium. Each medium was supplemented with 10% fetal bovine serum and 1% penicillin–streptomycin. All cell lines were incubated at 37°C in a humidified incubator with 5% CO_2_.

All cell lines were treated with 5 ng/ml of recombinant human TGF-β1 (Pepro Tech Inc., Rocky Hill, NJ, USA), 100 nM (MzChA-1 and GEM-resistant MzChA-1) or 300 nM (TFK-1 and GEM-resistant TFK-1) of the histone deacetylase inhibitor vorinostat (Selleckchem, Houston, TX, USA), or dimethyl sulfoxide (as a control) for 72 h. In the experiment with TGF-β1, each medium was changed to serum-free medium 24 h after passage to preclude the influence of TGF-β1 in serum. The TGF-β1 and vorinostat were added to each cell line after the change of medium. The antibodies described in S2 Table were used for western blot analysis, immunohistochemistry, and immunocytochemistry.

### Establishment of GEM-resistant MzChA-1 (MzChA-1_GR) and TFK-1 (TFK-1_GR) clones

The GEM-resistant MzChA-1 and TFK-1 cell lines were established through exposure to increasing concentrations of GEM (from 0.2 ng/μl to 80 ng/μl for MzChA-1, and from 0.2 ng/μl to 40 ng/μl for TFK-1) over 3 months. After confirming that these cell lines attained more resistance to GEM than the parent cell lines, the MzChA-1_GR and TFK-1_GR cell clones were established by limiting dilution.

### Assay of HDAC activity

The MzChA-1 cells were cultured with or without 5 ng/ml TGF-β1 and 300 nM vorinostat for 72 h. Then, nuclear proteins were extracted with the Nuclear Extract Kit (Active Motif, Carlsbad, CA, USA). Subsequently, the activity of HDAC was measured with the HDAC activity assay kit (BioVision, Palo Alto, USA). In this experiment, we used HeLa nuclear extract as a positive control and distilled water as a negative control. All procedures were conducted according to the manufacturer’s recommendations.

### Immunocytochemistry

Cells were fixed with 4% paraformaldehyde for 15 min at room temperature, permeabilized with 0.1% Triton X-100, and blocked with 1% BSA for 10 min at room temperature. Subsequently, the cells were stained with the indicated antibodies.

### Quantitative reverse transcription-polymerase chain reaction

Total RNA was isolated from cultured cells using Trizol reagent (Invitrogen, Carlsbad, CA, USA). Complementary DNA was synthesized from 3.0 μg of total RNA with the SuperScript first-strand synthesis system (Invitrogen) according to the manufacturer’s protocol. Quantitative real-time polymerase chain reactions (qRT-PCRs) were conducted with the LightCycler-Fast-Start DNA Master SYBR Green I kit (Roche Applied Science, Indianapolis, IN, USA) with gene-specific oligonucleotide primers, as shown in S3 Table. Amplifications were performed in triplicate using the LightCycler System (Roche Applied Science) in accordance with the manufacturer’s protocol. A melting curve analysis was performed to distinguish specific products from non-specific products and primer dimers. The relative expression was calculated as the ratio of specific mRNA to endogenous β-actin (ACTB) mRNA in each sample. All qRT-PCRs were performed in triplicate, and the results were presented as the mean ± standard deviation.

### Growth inhibition assays with GEM therapy

Each cell line (5×10^3^ cells/well) was seeded in a 96-well plate and incubated for 24 h. Then, the medium was changed to serum-free medium. Subsequently, the cells were exposed to GEM (1-120ng/mL) with the indicated drugs (TGF-β1, vorinostat, or dimethyl sulfoxide) for 72h. These assays were repeated at least three times, and similar results were obtained each time. The proportion of MTT (3-(4,5-Dimethylthiazol-2-yl)-2,5-diphenyltetrazolium bromide)-positive cells incubated without drugs was defined as 100% viability.

### Transfection of small interfering RNA (siRNA)

We used TGF-β siRNA (Invitrogen) and scrambled oligonucleotide siRNA (Sc) as a negative control. We performed siRNA transfection with Lipofectamine RNAiMAX (Invitrogen), according to the manufacturer’s protocols [28]. Transfection efficiency was confirmed by qRT-PCR.

### Western blot analysis

Western blot analysis was performed as described previously [28]. Briefly, a whole cell lysate was extracted from BTC cells with RIPA Buffer (Thermo Fisher Scientific, Inc., Rockford, IL, USA) and nuclear proteins were extracted with the EpiQuick Nuclear Extraction Kit I (Epigentek Group Inc., Brooklyn, NY, USA), according to the manufacturer’s protocol. Aliquots (12 μg) of total or nuclear proteins were electrophoresed on sodium dodecyl sulfate-polyacrylamide gels containing 10% Tris–HCl (Bio-Rad Laboratories Inc., Hercules, CA, USA). The separated proteins were transferred to polyvinylidene difluoride membranes (Millipore) and incubated with each primary antibody overnight at 4°C.

### Chromatin immunoprecipitation (ChIP)

The ChIP assay was performed with using a ChIP-IT Express Enzymatic kit (Active Motif) as previously described [29]. Briefly, 1 × 10^7^ cells were cross-linked with 1% formaldehyde for 10 min at room temperature and quenched by adding glycine. To harvest chromatin-DNA complexes, the cell lysate was treated with an enzymatic shearing kit (Active Motif), according to the manufacturer's specifications. For immunoprecipitation, 120 μg of chromatin-DNA complexes were incubated with Protein G magnetic beads linked to anti-SMAD4 antibody (10 μg, Cell Signaling Technology, Danvers, Massachusetts, USA), or anti-IgG antibody (10 μg, Active Motif). Eluates were used as templates for PCR. Equal amounts of anti-IgG or pre-immune chromatin-DNA complex were used as controls. The primer sets used for PCR amplification were: SNAI1: 5′-GCTGTCACACCCGGCACCAAG-3′ and 5′-GGCGGCTTGAAATGCCACGG-3′, SNAI2: 5′-ATGCGTGTGAAGTGCTTAGCATAGT-3′ and 5′-CACTCAGTGCCCAACAGTGTGT-3′, ZEB1: 5′-TTTCGGGAAGTTAAAATGTTTG-3′ and 5′-ATCCTGCTTCATCTGCCTGA-3′, ZEB2: 5′-TACGCCTGCGCTGTGACCTA-3′ and 5′-ACTCACTGGACCCGCCTCAG-3′, and TWIST: 5′-AGTCTCCTCCGACCGCTTCCTG-3′ and 5′-CTCCGTGCAGGCGGAAAGTTTGG-3′.

### Mouse xenograft tumor model

The MzChA-1 or MzChA-1_GR cells (1.0 × 10^6^ cells per mouse) were injected into the subcutaneous tissue on the back of five- to six-week-old male nude mice (CLEA Japan Inc., Tokyo, Japan), and tumors were allowed to develop. When the tumors grew to the volume of 200 mm^3^, GEM (125 mg/kg, once a week) with or without vorinostat (60 mg/kg, 5 consecutive days per week) was administered by intraperitoneal injection. To investigate the effect of vorinostat on survival time, treatment was continued until appropriate euthanasia. Resected specimens of tumors were used for immunohistochemistry to investigate the expression of SMAD4.

### Immunohistochemistry

Immunohistochemical studies on 10 resected tumor specimens from the mice xenografted with MzChA-1_GR cells. Briefly, formalin-fixed, paraffin-embedded tissues were deparaffinized, boiled for antigen retrieval, incubated with a SMAD4 antibody for 1 hour at room temperature, then visualized with avidin–biotin complex reagents (Vector Laboratory Inc., Burlingame, CA, USA) and diaminobenzidine. All sections were counterstained with hematoxylin. All immunohistochemical studies were evaluated in serial sections with each antibody.

### Ethical statements

All animal experiments were carried out in strict accordance with the Institutional Animal Care and Use Committee at the Osaka University (approval number: 20–087), and studies were conducted in compliance with institutional guidelines. In the animal experiments, all invasive treatments such as xenograft transplantation, gavage, and euthanasia were performed under general anesthesia with sevoflurane. The mice were euthanized at the onset of clinical signs such as severe cachexia, significant weight loss, or inactivity.

### Statistical analysis

The data are presented as the mean ± standard deviation (SD) from at least three independent experiments. Statistical analysis was performed using Student’s t-test or Fisher’s exact test for categorical data. The unpaired Student’s t-test was used to examine differences in growth inhibitory effects *in vitro*. *P*-values < 0.05 were considered statistically significant.

## Results

### Establishment of Mz-ChA-1_GR and TFK-1_GR cell lines

We established the GEM-resistant cell lines, MzChA-1_GR and TFK-1_GR, to investigate the effect of HDAC inhibitors on chemoresistance. The MzChA-1_GR cells were resistant to GEM (IC_50_ for GEM > 100 ng/mL, *P* < 0.01 versus the parent MzChA-1 cells, S1A Fig) and TFK-1_GR cells were resistant to GEM (IC_50_ for GEM > 100 ng/mL, *P* < 0.01 versus the parent TFK-1 cells, S1B Fig). These GEM-resistant cells had spindle-like shapes.

### Microarray analysis

A microarray analysis was performed using the TORAY 3D-Gene^®^ to compare the expression profiles of MzChA-1 cells with those of MzChA-1_GR cells and investigate the factors related to epigenetics. The results showed that the expression of class I HDACs (HDAC-1, HDAC-2, HDAC-3, and HDAC-8) were higher in MzChA-1_GR cells than parent MzChA-1 cells (Table 1). Vorinostat inhibits these class I HDACs, so we considered that vorinostat might be effective in chemoresistant BTC cells.

**Table 1:** The expression of class I HDACs in MzChA-1 (parent) and MzChA-1_GR cells, determined by microarray analysis.

	Parent	GR1	GR2	GR3	Fold change
*HDAC1*	262	362	336	336	1.32
*HDAC2*	110	132	107	150	1.23
*HDAC3*	34	41	35	37	1.11
*HDAC8*	63	88	73	80	1.27

Abbreviations: GR, gemcitabine-resistant; HDAC, histone deacetylase

### The mRNA expression of class I HDACs and HDAC activity in MzChA-1 and MzChA-1_GR cells

**Fig 1:**
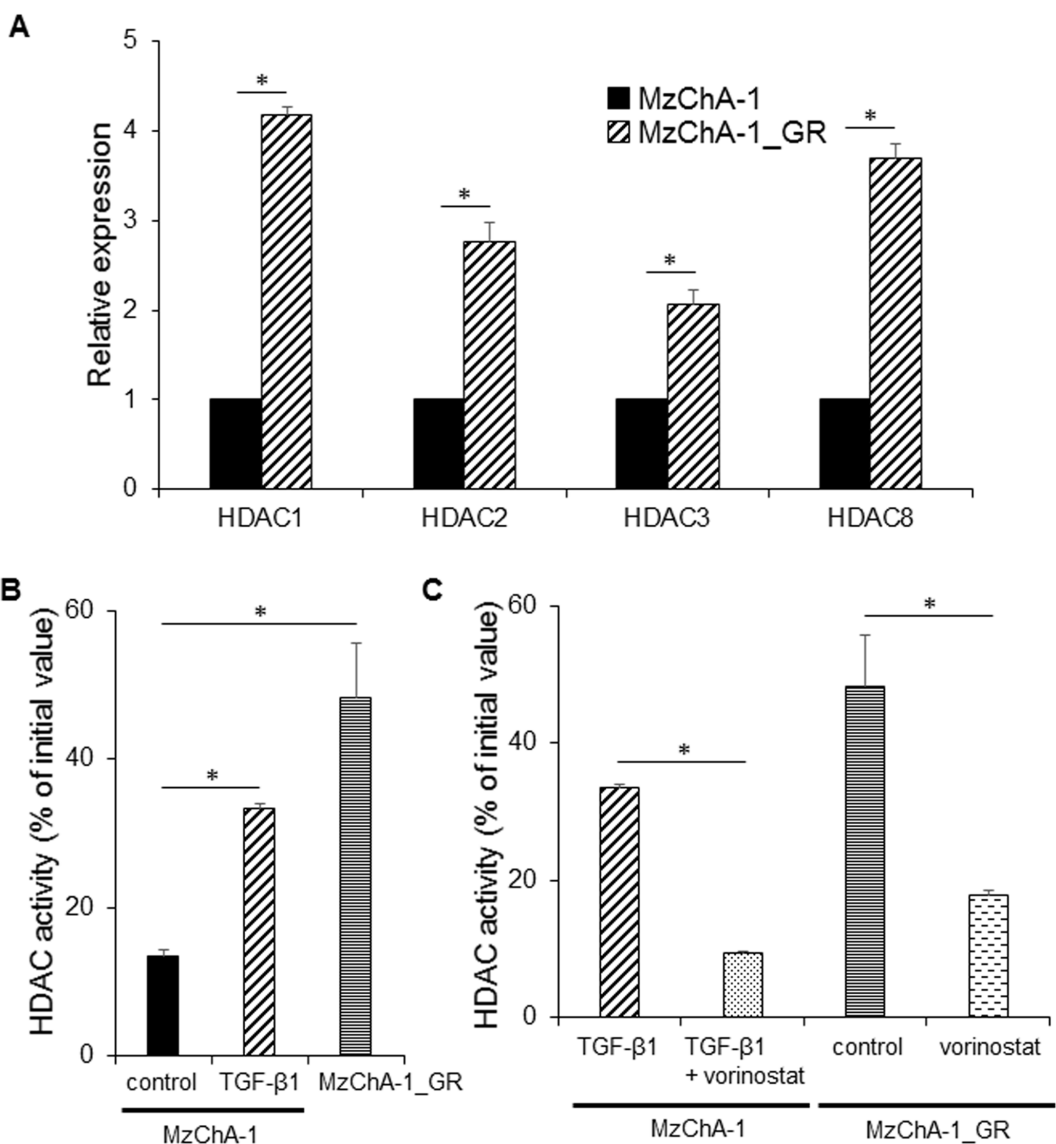
The mRNA expression of class I HDACs and HDAC activity in MzChA-1 and MzChA-1_GR cells. Values represent the mean ± S.D. *P < 0.05. All experiments were conducted at least three times. (A) Comparison of the expression of class I HDACs in MzChA-1 and MzChA-1_GR cells by qRT-PCR. (B) The effect of TGF-β1 on HDAC activity in MzChA-1 cells and the HDAC activity in MzChA-1_GR cells, activity was measured with the HDAC activity assay kit. (C) The changes in HDAC activity in MzChA-1 and MzChA-1_GR cells treated with TGF-β1 and vorinostat. In the experiments for panels (B) and (C), the cells were incubated with or without 5 ng/ml TGF-β1 or 100 nM vorinostat for 72 h.

To validate the microarray analysis results, qRT-PCR for class I HDACs was performed for MzChA-1 and MzChA-1_GR cells. All class I HDACs showed higher expression in MzChA-1_GR cells compared with MzChA-1 cells (*P*<0.05, Fig 1A). The HDAC activity was significantly higher in MzChA-1 cells exposed to TGF-β1 for 72 h and MzChA-1_GR cells compared with parent MzChA-1 cells (*P*<0.05, Fig 1B). In addition, vorinostat suppressed the HDAC activity in both MzChA-1 cells exposed to TGF-β1 and MzChA-1_GR cells (*P*<0.05, Fig 1C).

### The relationship between TGF-β1 expression and chemoresistance

**Fig 2:**
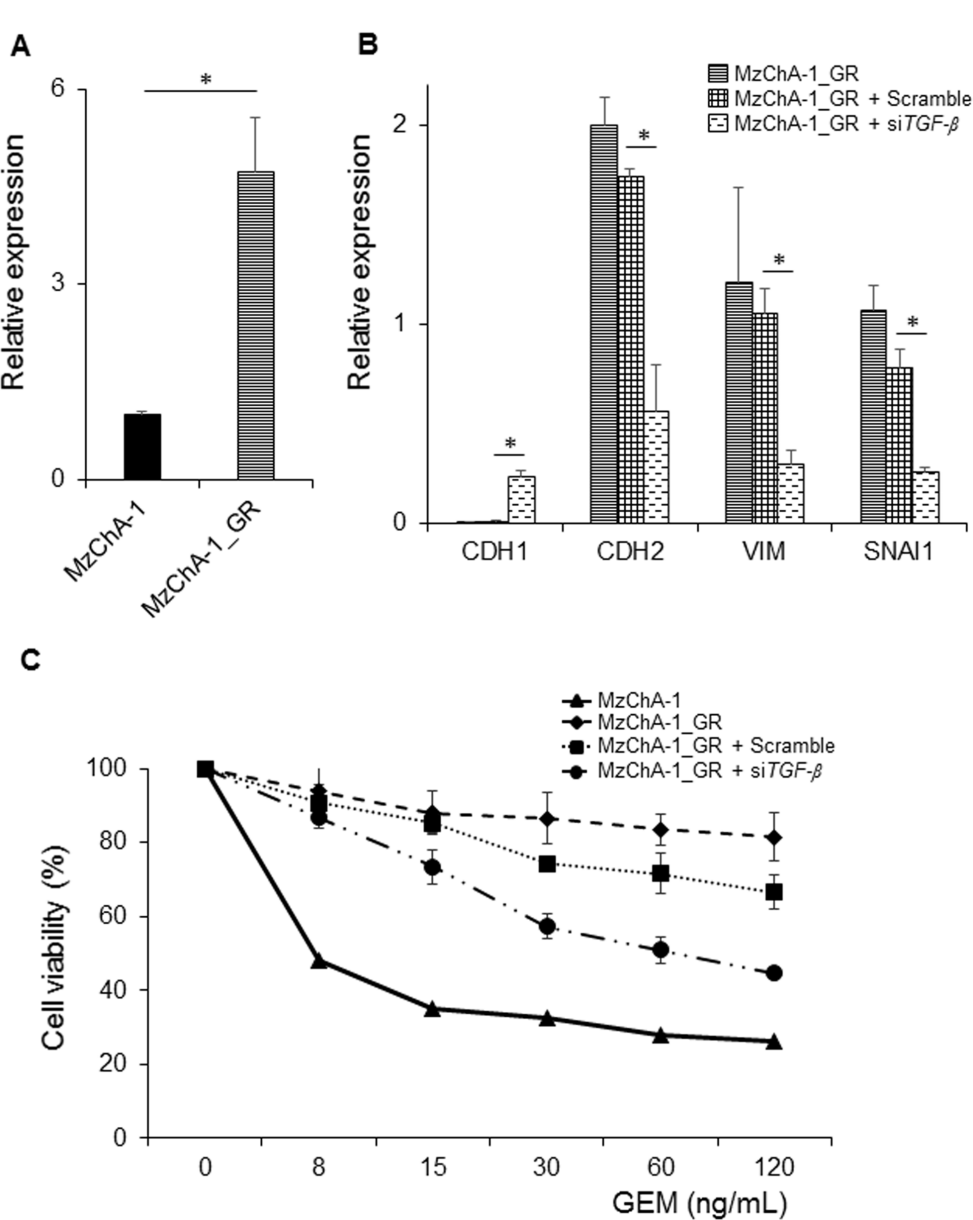
The effect of TGF-β1 knockdown using TGF-β small interfering RNA (siRNA) on EMT and chemoresistance in MzChA-1_GR cells. MzChA-1_GR cells were transfected with scrambled oligonucleotide siRNA (negative control) or TGF-β1 siRNA. All experiments were conducted at least three times. Values represent the mean ± S.D. *P < 0.05. (A) Comparison of the expression of TGF-β1 in MzChA-1 and MzChA-1_GR cells. (B) The changes of EMT-related mRNA expression as a result of TGF-β siRNA transfection in MzChA-1_GR cells. (C) The effect of TGF-β siRNA transfection on chemoresistance in MzChA-1_GR cells. Growth inhibition assays were performed for transfected and non-transfected cells treated with GEM.

To clarify the significance of TGF-β1 in EMT and chemoresistance, we investigated the expression of TGF-β1 in parent MzChA-1 and MzChA-1_GR cells. The results showed that the expression level of TGF-β1 was significantly higher in MzChA-1_GR cells than parent MzChA-1 cells (Fig 2A). Then, we transfected with si*TGF-β* to investigate whether EMT and chemoresistance could be suppressed by TGF-β knock down in MzChA-1_GR cells. The result showed that MzChA-1_GR cells transfected with si*TGF-β* had increased the mRNA expression of CDH1 and decreased the mRNA expression of CDH2, vimentin, and SNAI1 (Fig 2B). Moreover, transfection with si*TGF-β* attenuated chemoresistance in MzChA-1_GR cells (Fig 2C). These results suggested the significance of TGF-β in EMT and chemoresistance in BTC.

### The effect of vorinostat on TGF-β1-induced EMT and chemoresistance

**Fig 3:**
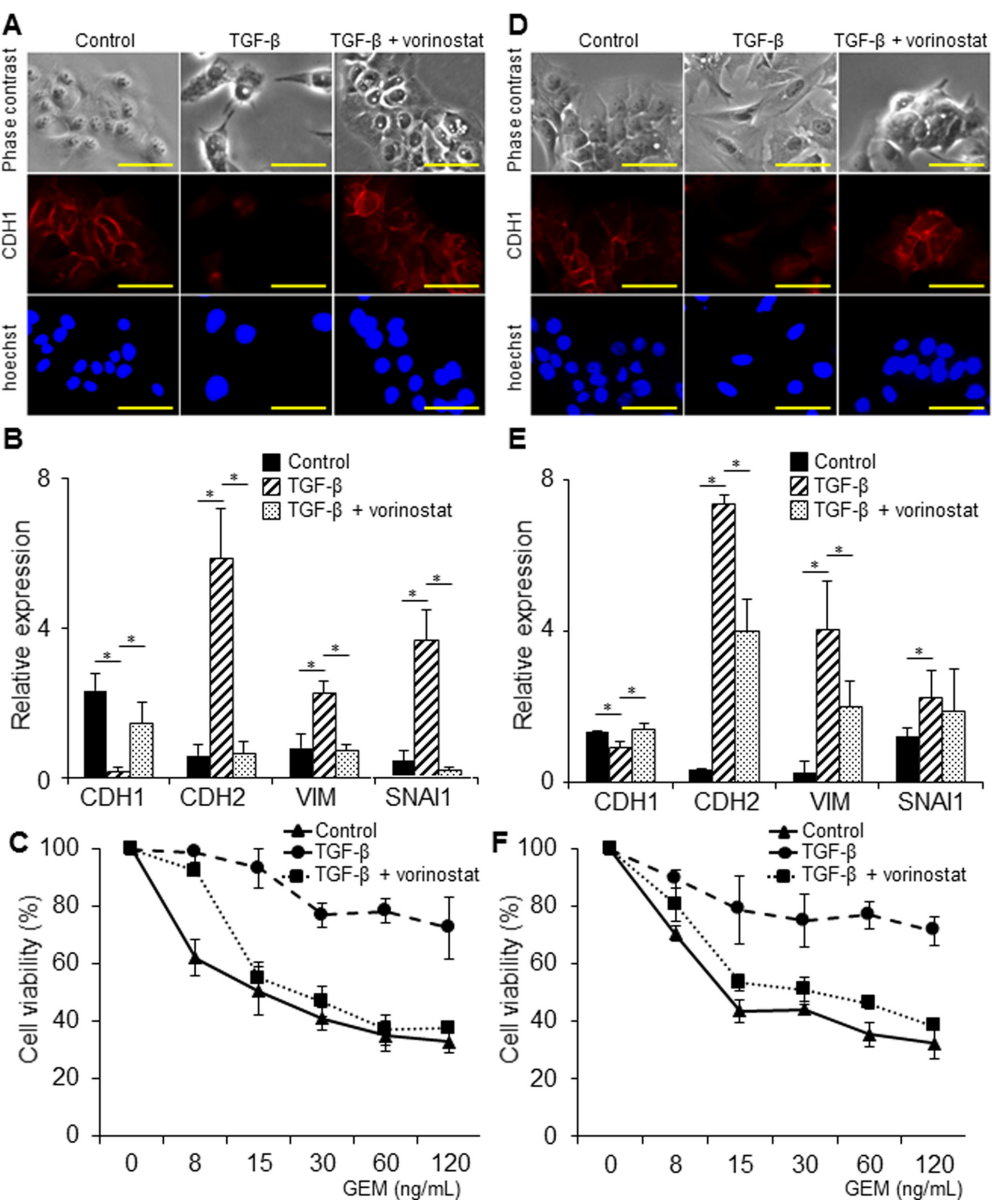
Influence of vorinostat on TGF-β1-induced EMT and chemoresistance in BTC cell lines. In each experiment, the cells were incubated with or without 5 ng/ml of TGF-β1 and 100 nM of vorinostat for MzChA-1 cells and 300 nM of vorinostat for TFK-1 cells for 72 h. Values represent the mean ± S.D. *P < 0.05. Scale bars: 100 μm. All experiments were conducted at least three times. (A) Representative cell morphological changes induced by TGF-β and vorinostat in MzChA-1 cells. Immunofluorescence for CDH1 (red) was performed in MzChA-1 cells. Nuclear staining (blue) was performed with Hoechst. (B) The effect of vorinostat on the EMT-related mRNAs expression in MzChA-1 cells. (C) The effect of vorinostat on chemoresistance induced by TGF-β1 exposure in MzChA-1 cells. The growth inhibition assays were performed for MzChA-1 cells treated with GEM. (D) Representative cell morphological changes induced by TGF-β and vorinostat in TFK-1 cells. Immunofluorescence for CDH1 (red) was performed in TFK-1 cells. Nuclear staining (blue) was performed with Hoechst. (E) The effect of vorinostat on the EMT-related mRNAs expression in TFK-1 cells. (F) The effect of vorinostat on chemoresistance induced by TGF-β1 exposure in TFK-1 cells. Growth inhibition assays were performed for TFK-1 cells treated with GEM.

We investigated the effect of vorinostat on TGF-β1-induced EMT and chemoresistance in BTC cells. In MzChA-1 cells, TGF-β1 caused morphological changes of the cells, from valvate-like shapes to spindle-like shapes. Moreover, TGF-β1 down-regulated the expression of CDH1 as determined by immunofluorescence staining (Fig 3A). In contrast, vorinostat prevented these changes caused by TGF-β1 (Fig 3A). Furthermore, TGF-β1 decreased the mRNA expression of CDH1 and increased the mRNA expression of CDH2, vimentin (VIM), and SNAI1. In contrast, vorinostat inhibited the changes in mRNA expression caused by TGF-β1 (*P*<0.05, Fig 3B). Additionally, vorinostat attenuated GEM resistance induced by TGF-β1 (*P*<0.05, Fig 3C). In TFK-1 cells, vorinostat prevented morphological changes and the down-regulation of CDH1 caused by TGF-β1 (Fig 3D). Vorinostat also inhibited the changes in mRNA expression related to EMT (*P*<0.05, Fig 3E), and attenuated chemoresistance to GEM caused by TGF-β1 (*P*<0.05, Fig 3F). Thus, vorinostat suppressed EMT and attenuated chemoresistance caused by TGF-β1.

### The influence of vorinostat on MzChA-1_GR and TFK-1_GR cells

**Fig 4:**
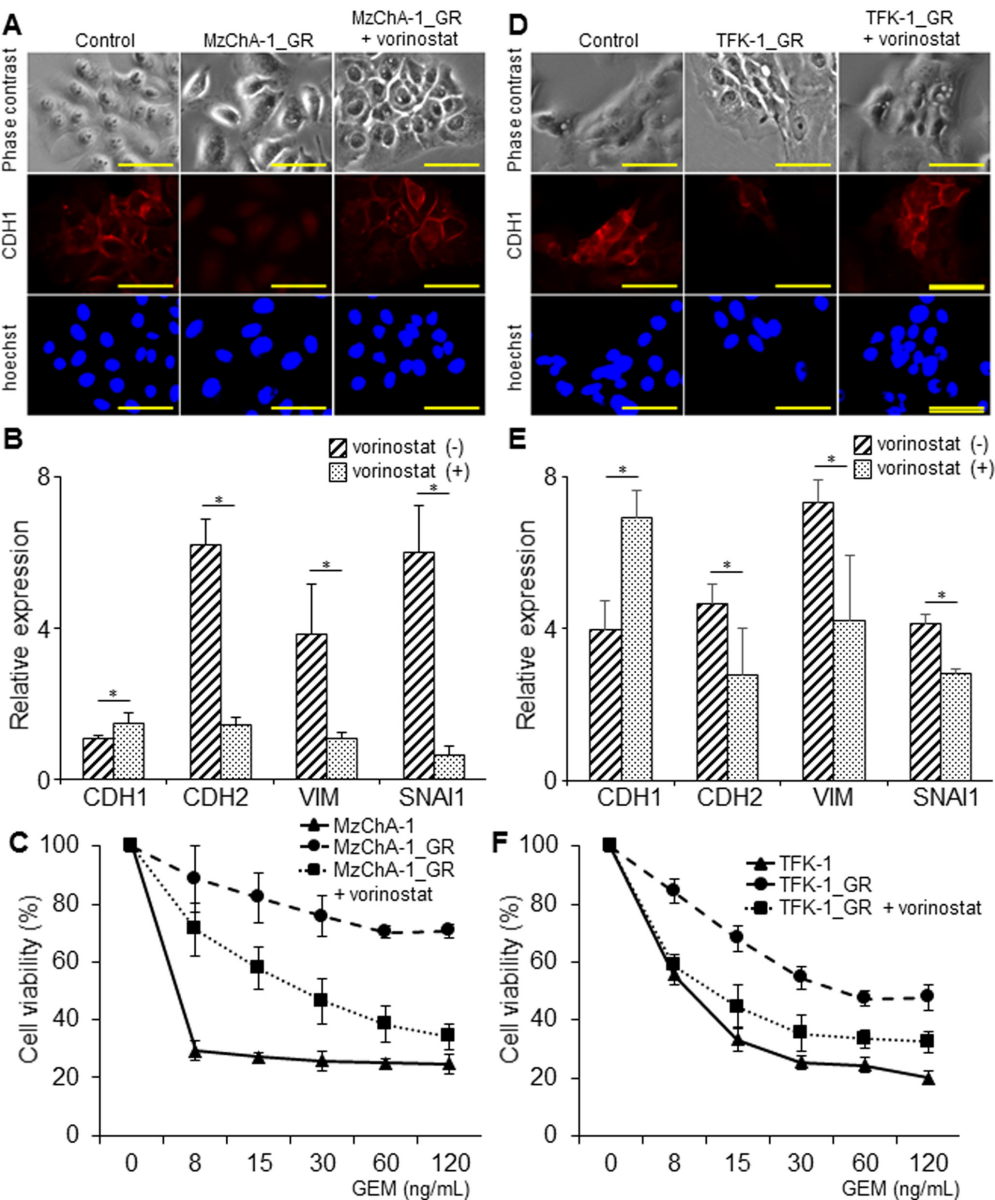
Influence of vorinostat on chemoresistant BTC cells. In each experiment, MzChA-1_GR cells were incubated with or without 100 nM vorinostat and TFK-1_GR cells with or without 300nM vorinostat for 72 h. Values represent the mean ± S.D. *P < 0.05. Scale bars: 100 μm. All experiments were conducted at least three times. (A) Representative morphological change induced by vorinostat in MzChA-1_GR cells. Immunofluorescence for CDH1 (red) was performed in MzChA-1 cells. Nuclear staining (blue) was performed with Hoechst. (B) The effect of vorinostat on the EMT-related mRNAs expression in MzChA-1_GR cells. (C) The effect of vorinostat on chemoresistance in MzChA-1_GRcells. Growth inhibition assays were performed for MzChA-1_GR cells treated with GEM. (D) Representative morphological changes induced by vorinostat in TFK-1_GR cells. Immunofluorescence for CDH1 (red) was performed in TFK-1_GR cells. Nuclear staining (blue) was performed with Hoechst. (E) The effect of vorinostat on the EMT-related mRNAs expression in TFK-1_GR cells. (F) The effect of vorinostat on chemoresistance in TFK-1_GR cells. Growth inhibition assays were performed for TFK-1_GR cells treated with GEM.

In MzChA-1_GR cells, vorinostat changed the morphology from spindle-like shapes to valvate-like shapes and enhanced the staining of CDH1, as determined by immunofluorescence staining (Fig 4A). The CDH1 mRNA expression tended to increase, and CDH2, VIM, and SNAI1 were significantly decreased by vorinostat (*P*<0.05, Fig 4B). In addition, vorinostat attenuated GEM chemoresistance in MzChA-1_GR cells (*P*<0.05, Fig 4C).

In TFK-1_GR cells, vorinostat changed the morphology and enhanced the staining of CDH1, as measured by immunofluorescence staining similar to MzChA-1_GR cells (Fig 4D). In addition, vorinostat increased the expression of CDH1 mRNA, and decreased the mRNA expression of CDH2, VIM, and SNAI1 (*P*<0.05, Fig 4E). GEM resistance was also attenuated by vorinostat in TFK-1_GR cells (*P*<0.05, Fig 4F). These results suggested that vorinostat suppressed EMT and attenuated chemoresistance in GEM resistant BTC cells.

### The effect of vorinostat on SMAD4 nuclear translocation in MzChA-1 cells

**Fig 5:**
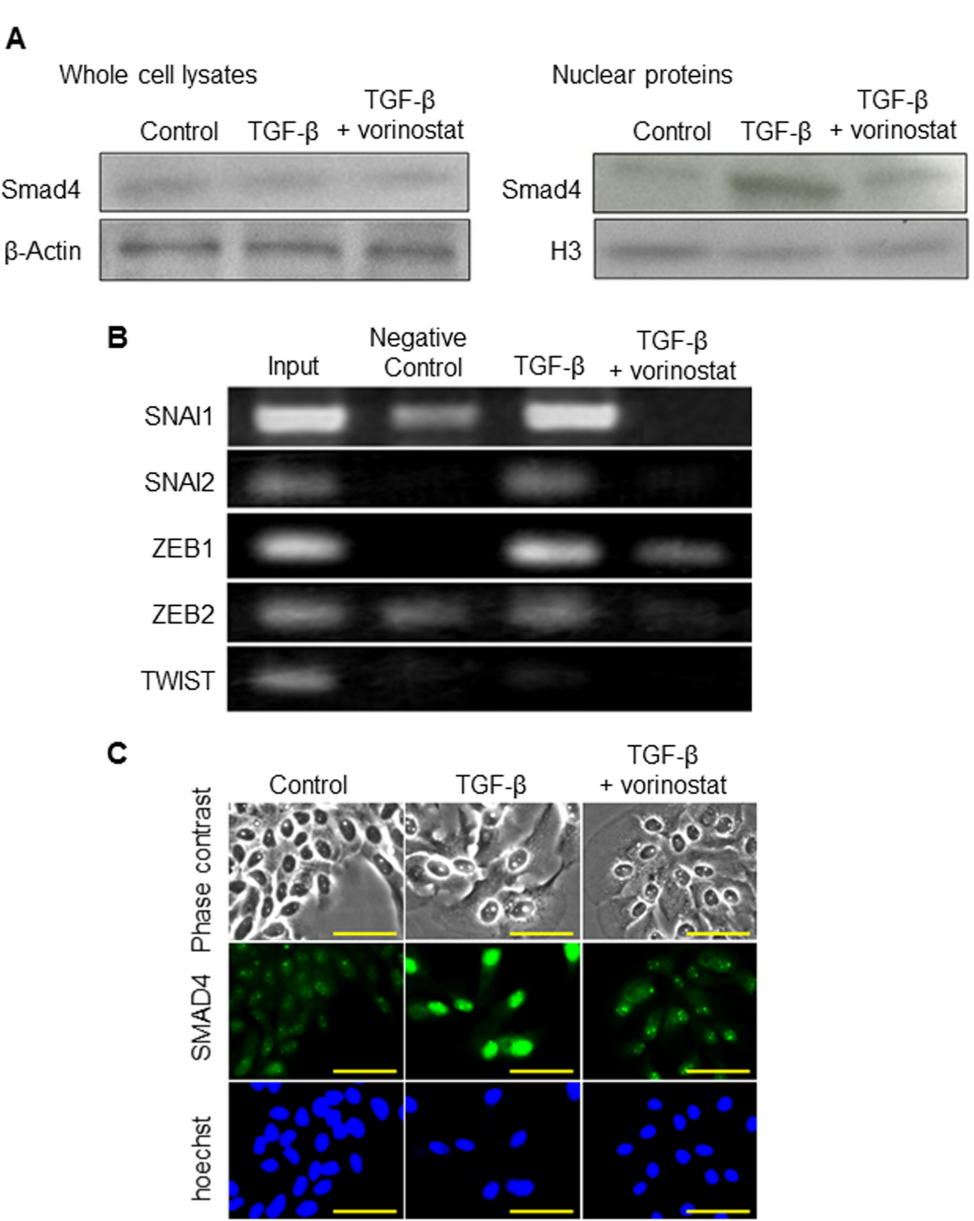
Inhibition of the nuclear translocation of SMAD4 by vorinostat in MzChA-1 cells. In each experiment, cells were incubated with or without 5 ng/ml of TGF-β1 and 100 nM of vorinostat for 72 h. Scale bars: 100 μm. All experiments were conducted at least three times. (A) The change in SMAD4 expression in whole cell lysates (left panel) and in the nucleus (right panel) caused by TGF-β and vorinostat. (B) ChIP assay showing expression of SNAI1, SNAI2, ZEB1, ZEB2, and TWIST, which are regulatory elements of CDH1. (C) Immunofluorescence of SMAD4 (green) was performed in MzChA-1 cells. The effect of TGF-β1 and vorinostat on SMAD4 nuclear translocation were investigated. Nuclear staining (blue) was performed with Hoechst.

In our previous study, SMAD4 was the key molecule regulating TGF-β1-induced EMT [10]. Therefore, we investigated the effect of vorinostat on the expression of SMAD4 by western blot analysis. In whole cell lysates, there was no significant difference in the expression of SMAD4 protein between the TGF-β1 group and the TGF-β1 plus vorinostat group (Fig 5A, left). However, TGF-β1 upregulated the expression of the SMAD4 nucleoprotein compared with the control, and vorinostat inhibited this upregulation of the SMAD4 nucleoprotein caused by TGF-β1 (Fig 5A, right). Furthermore, we used the ChIP method to analyze the molecular mechanism of SMAD4. While TGF-β1 enhanced the binding affinity of SMAD4 to the CDH1-related transcriptional factors SNAI1, SNAI2, ZEB1, ZEB2, and TWIST, vorinostat inhibited the TGF-β1-enhanced binding affinity of SMAD4 to these transcriptional factors (Fig 5B).

Immunocytochemistry demonstrated that SMAD4 stained strongly in the nuclei after treatment with TGF-β1 (Fig 5C). In contrast, SMAD4 was weakly stained in the nuclei after treatment with TGF-β1 plus vorinostat (Fig 5C). Based on these results, vorinostat regulated EMT through inhibition of SMAD4 nuclear translocation.

### The effect of vorinostat on the TGF-β signaling factors, SMAD2, SMAD3, and Jun N-terminal kinase (JNK)

**Fig 6:**
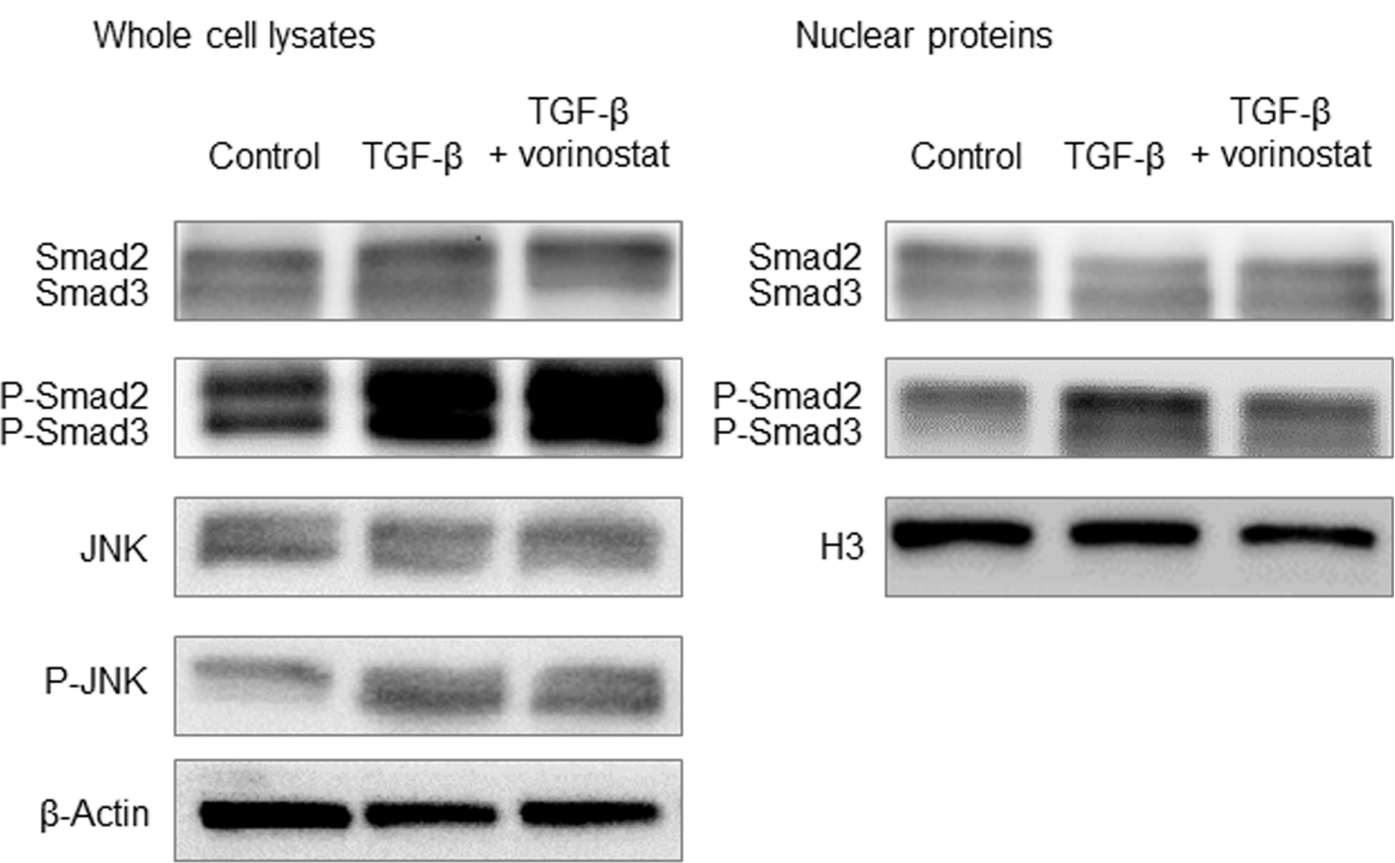
The effect of TGF-β1 and vorinostat on SMAD2, SMAD3, and JNK in MzChA-1. In each experiment, cells were incubated with or without 5 ng/ml of TGF-β1 and 100 nM of vorinostat for 72 h. All experiments were conducted at least three times. The changes in SMAD2, p-SMAD2, SMAD3, and p-SMAD3 expression in whole cell lysates (left panel) and in the nucleus (right panel) are shown. The expression of JNK and p-JNK in whole cell lysates are also shown (left panel).

We also investigated the effect of vorinostat on SMAD2 and SMAD3 because they are closely related to SMAD4 nuclear translocation in TGF-β signaling. We found TGF-β1 induced SMAD2 and SMAD3 phosphorylation in both whole cell lysates and nuclear proteins (Fig 6). In addition, vorinostat inhibited TGF-β1-induced nuclear translocation of phosphorylated SMAD2 (p-SMAD2) and SMAD3 (p-SMAD3); while, vorinostat had no effect on the nuclear translocation of SMAD2 and SMAD3 (Fig 6). These results suggest that vorinostat can inhibit nuclear translocation of p-SMAD2, p-SMAD3, and SMAD4 at the same time. We also investigated the activation of JNK because recent reports showed that JNK activation plays a central role in TGF-β mediated EMT. The result showed TGF-β1 upregulated the expression of phosphorylated JNK; however, vorinostat had no effect on JNK (Fig 6). Accordingly, the effect of vorinostat on EMT seems to be independent of the JNK pathway in BTC.

### The effect of vorinostat on the mouse xenografted tumor model

**Fig 7:**
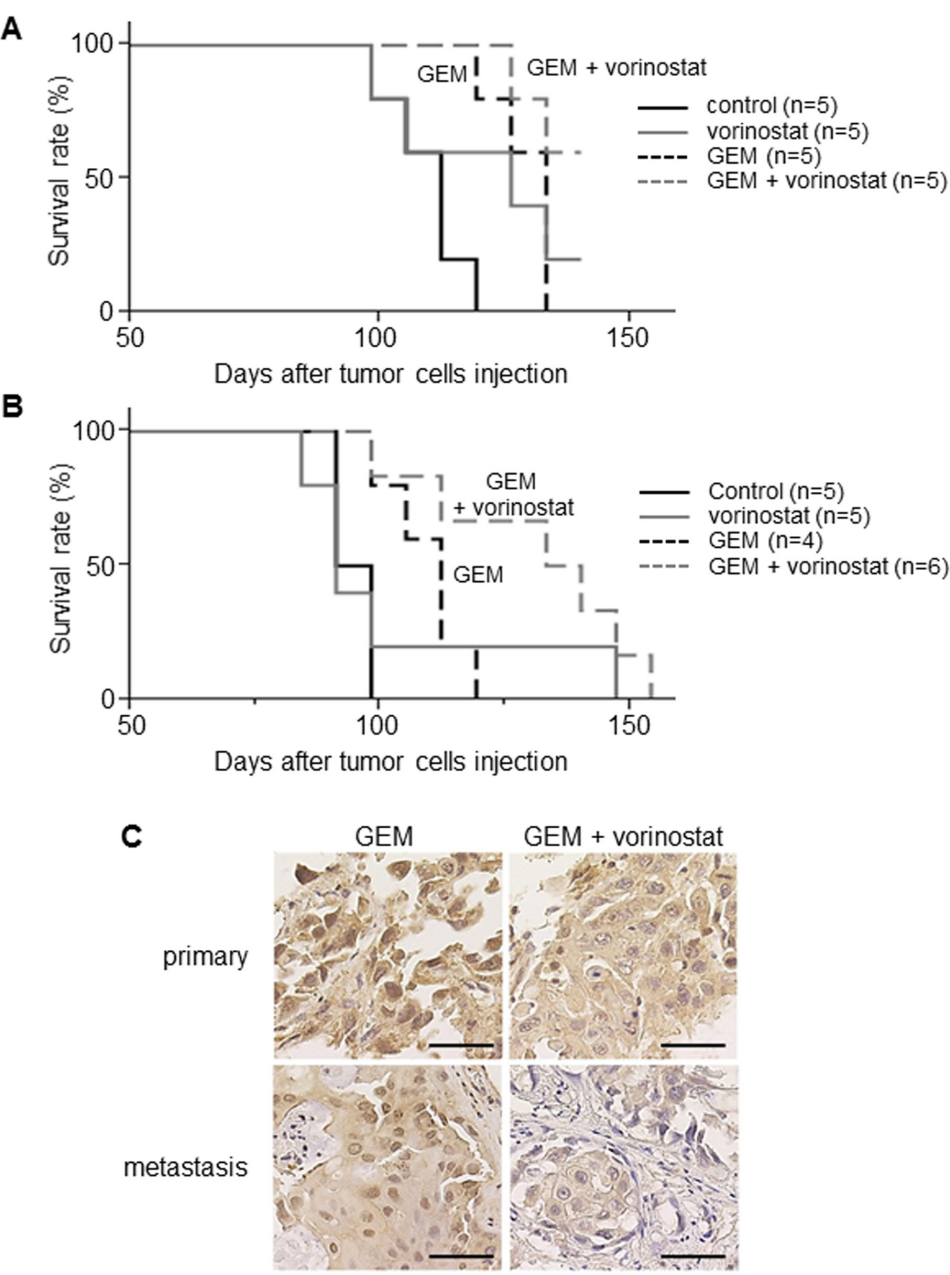
*In vivo* analysis of mice xenografted with MzChA-1 and MzChA-1_GR cells. NOD/SCID mice xenografted with MzChA-1 and MzChA-1_GR cells were employed as *in vivo* models. When the tumor volume was over 200 mm^3^, the mice were treated with the indicated drugs (GEM [125 mg/kg, once a week] and/or vorinostat [60 mg/kg, 5 consecutive days per week]). The prognostic value was evaluated by the Kaplan–Meier method and a log-rank test. (A) Survival curve for mice xenografted with MzChA-1 cells. (B) Survival curve for mice xenografted with MzChA-1_GR cells. (C) Immunohistochemistry for SMAD4 on the resected tumor specimens of the mice xenografted with MzChA-1_GR cells.

To confirm the *in vitro* results, we evaluated the effect of vorinostat on mice xenografted with MzChA-1 or MzChA-1_GR cells. In the mice xenografted with MzChA-1 cells, combination therapy with vorinostat and GEM did not improve the survival time compared with a single administration of GEM (Fig 7A). In contrast, combination therapy with vorinostat and GEM improved the survival time compared with a single administration of GEM in the mice xenografted with MzChA-1_GR cells (log-rank test, *P*<0.01, Fig 7B).

Subsequently, we performed immunohistochemistry on the resected tumor specimens of the mice to evaluate the expression of SMAD4 *in vivo*. The result showed that SMAD4 was strongly stained in the nuclei of tumor cells when the mice were treated with a single administration of GEM. In contrast, SMAD4 was weakly stained in the nuclei of tumor cells when the mice were treated with GEM and vorinostat (Fig 7C). These results were the same in both primary and metastatic sites (pulmonary). Thus, vorinostat improved survival time in the mice xenografted with chemoresistant cells when they were treated with GEM.

## Discussion

In many cancers, several genes related to tumorigenesis and tumor progression are upregulated, and genes related to tumor suppression are downregulated. One of the factors that regulate this gene expression is posttranslational modification of the histones. Methylation and acetylation of histones are well known post-translational modifications [30–32]. Recently, much attention has been paid to the methylation and acetylation of histones for the treatment of cancers [33,34]. In this study, we focused on the ability of HDAC inhibitors to regulate EMT. We used vorinostat, which inhibits class I HDACs and is often used as a HDAC inhibitor in cancer clinical trials, as shown in S4 Table [35–39]. In several cancers, such as non-small cell lung cancer and breast cancer, combination chemotherapy with vorinostat was safe and showed potential for improving the efficacy [38,40].

We focused on SMADs, particularly SMAD4 which is a signaling factor of TGF-β1, as one of the mechanisms that vorinostat regulates EMT. It is often reported that TGF-β induces EMT and chemoresistance [10]. Activated type I TGF-β1 receptors phosphorylate SMAD2 and SMAD3, which form a heteromeric complex with SMAD4 [41,42]. This complex translocates to the nucleus and targets a variety of DNA binding proteins to regulate transcriptional responses [41,42]. Thus, transcriptional regulators of CDH1, such as SNAI, ZEB, and TWIST, are upregulated [42]. Then, CDH1 is downregulated and EMT occurs [43,44]. Thus, inhibiting nuclear translocation of SMAD2, SMAD3, and SMAD4 complexes could regulate TGF-β1-related EMT. First, we investigated whether vorinostat changes the mRNA expression of these SMADs by qRT-PCR. No changes in these SMADs were observed after exposure to vorinostat (S2 Fig). However, a few studies have shown aneffect of HDAC inhibitors on SMAD4 [22,23]. Kaimori et al. showed that histone deacetylase inhibition suppresses the TGF-β1-induced EMT in hepatocytes via inhibition of SMAD4 nuclear translocation [22]. Chung et al. showed that the HDAC inhibitor, m-carboxycinnamic acid, inhibited SMAD4 nuclear translocation in human pleural mesothelial cells [23]. However, there are no studies on the effect of HDAC inhibitors on SMAD4 nuclear translocation in any cancer. Our investigation showed that vorinostat inhibited SMAD4 nuclear translocation induced by TGF-β1 in BTC. We also showed that vorinostat inhibited nuclear translocation of p-SMAD2 and p-SMAD3. These results suggest that vorinostat inhibits nuclear translocation of the p-SMAD2, p-SMAD3, and SMAD4 complex.

To clarify whether vorinostat sufficiently inhibited EMT related to TGF-β signaling, we investigated the effect of vorinostat and si*TGF-β* transfection in MzChA-1_GR cells. We found vorinostat with si*TGF-β* transfection had no additional effect on EMT and chemoresistance *(data not shown)*. This result suggests that the effect of vorinostat is similar to transfection with siTGF-β in regulating EMT and chemoresistance.

*In vivo* experiments showed that vorinostat with GEM treatment improved the survival time of the mice xenografted with MzChA-1_GR cells compared with single administration of GEM. However, there was no significant change in tumor development between the vorinostat with GEM treatment group and GEM treatment group (S3 Fig). One of the reasons why vorinostat could improve the survival rate might be that vorinostat suppressed tumor metastasis. In fact, the tumors in metastatic sites tended to be large in the GEM treatment group compared with the vorinostat with GEM treatment group. Immunohistochemistry showed that SMAD4 was more strongly stained in the nuclei of cancer cells in the GEM treatment group compared with the vorinostat with GEM treatment group. These results suggest that vorinostat regulates tumor progression and metastasis by inhibiting nuclear translocation of SMAD4 *in vivo*.

Some studies reported that HDAC inhibitors have a similar function as TGF-β via activation of CDK inhibitors. One of the representative CDK inhibitors related to EMT is p21, and TGF-β induces p21 through a p53-independent mechanism [45]. The HDAC inhibitors are reported to activate p21 in breast cancer cells [46]. Several studies showed that p21 inhibited EMT [47]. Therefore, we investigated the change in p21 expression by TGF-β1 and vorinostat. The result showed that only TGF-β1 induced p21 expression (S4 Fig). In addition, another CDK inhibitor, p15, which is reported to be induced by TGF-β1 was similarly induced (S4 Fig).These results indicate the effect of vorinostat in regulating the activation of CDK inhibitors might differ depending on the kind of cancer. This issue is controversial and more investigation would be needed in other types of cancer.

We used vorinostat in this study; however, other HDAC inhibitors, such as trichostatin A, panobinostat, entinostat, and valproic acid have been used to regulate EMT, and some of them have been used in clinical trials [48–51]. In the future, these drugs will need to be tested to determine if they regulate EMT and chemoresistance, as suggested by the results of this study. In conclusion, the HDAC inhibitor, vorinostat, regulated EMT and chemoresistance in BTC via inhibition of nuclear translocation of SMAD4 *in vitro* and *in vivo*.

## Supporting Information

S1 Fig Growth inhibition assays for GEM in parent cell lines and GEM-resistant cell lines. Comparison of chemoresistance in parent cell lines and established GEM-resistant cell lines by growth inhibition assays. Values represent the mean ± S.D. *P < 0.05. (A) Growth inhibition assay in MzChA-1 and MzChA-1_GR cells. (B) Growth inhibition assay in TFK-1 and TFK-1_GR cells.
(TIF)

S2 Fig The effect of TGF-β and vorinostat on the SMAD2, SMAD3, and SMAD4 mRNAs expression in MzChA-1 cells. Each cell line was cultured with or without 5 ng/ml of TGF-β1 and 100 nM of vorinostat for 72 h. Then, SMAD2, SMAD3, and SMAD4 were assessed by qRT-PCR. Values represent the mean ± S.D. All experiments were conducted at least three times.
(TIF)

S3 Fig Tumor progression in the mice xenografted with MzChA-1_GR cells. Tumor progression was assessed by tumor volume. Values represent the mean ± S.D.
(TIF)

S4 Fig The effect of TGF-β and vorinostat on the expression of p15 and p21 in MzChA-1 cells. Each cell line was cultured with or without 5 ng/ml of TGF-β1 and 100 nM of vorinostat for 72 h. Then, p15 and p21 were assessed by qRT-PCR. Values represent the mean ± S.D. *P < 0.05. All experiments were conducted at least three times.
(TIF)

S1 Table Cytokines associated with EMT (Table A). References for S1A Table (Table B). (DOCX)

S2 Table Antibodies used in western blot analysis, immunohistochemistry, and immunocytochemistry. (DOCX)

S3 Table Primers used in qRT-PCR. (DOCX)

S4 Table Clinical trials involving HDAC inhibitors (Table A). References for S4A Table (Table B). (DOCX)
