# Supplementary figures and images for: A Histone Deacetylase Inhibitor Suppresses Epithelial-Mesenchymal Transition and Attenuates Chemoresistance in Biliary Tract Cancer
Source: PLoS One. 2016 Jan 4;11(1):e0145985. doi: 10.1371/journal.pone.0145985 (PMC4699768; doi:10.1371/journal.pone.0145985)

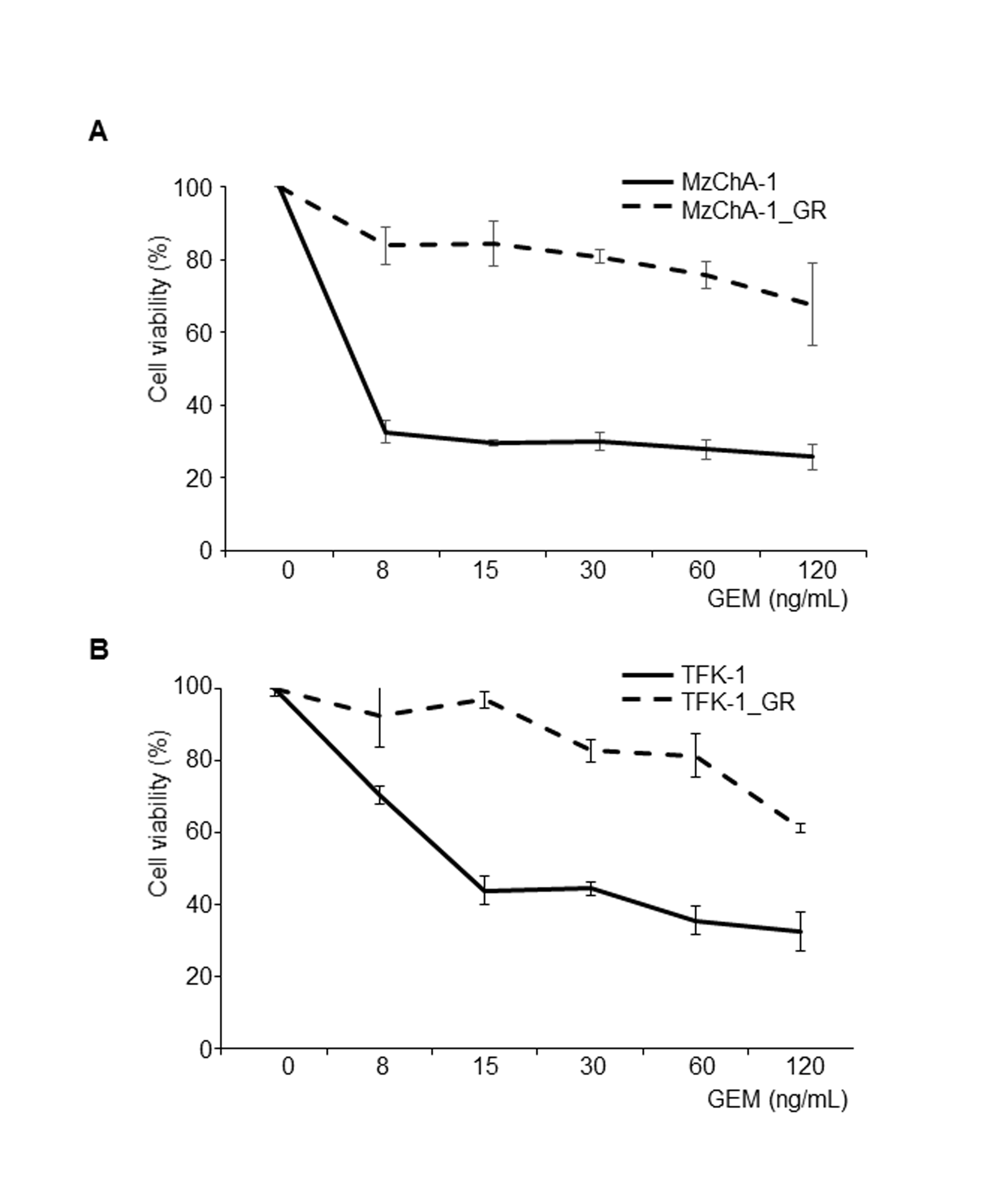

Supplement: S1 Fig — Comparison of chemoresistance in parent cell lines and established GEM-resistant cell lines by growth inhibition assays. Values represent the mean ± S.D. *P < 0.05. (A) Growth inhibition assay in MzChA-1 and MzChA-1_GR cells. (B) Growth inhibition assay in TFK-1 and TFK-1_GR cells. (TIF) [file pone.0145985.s001.TIF]

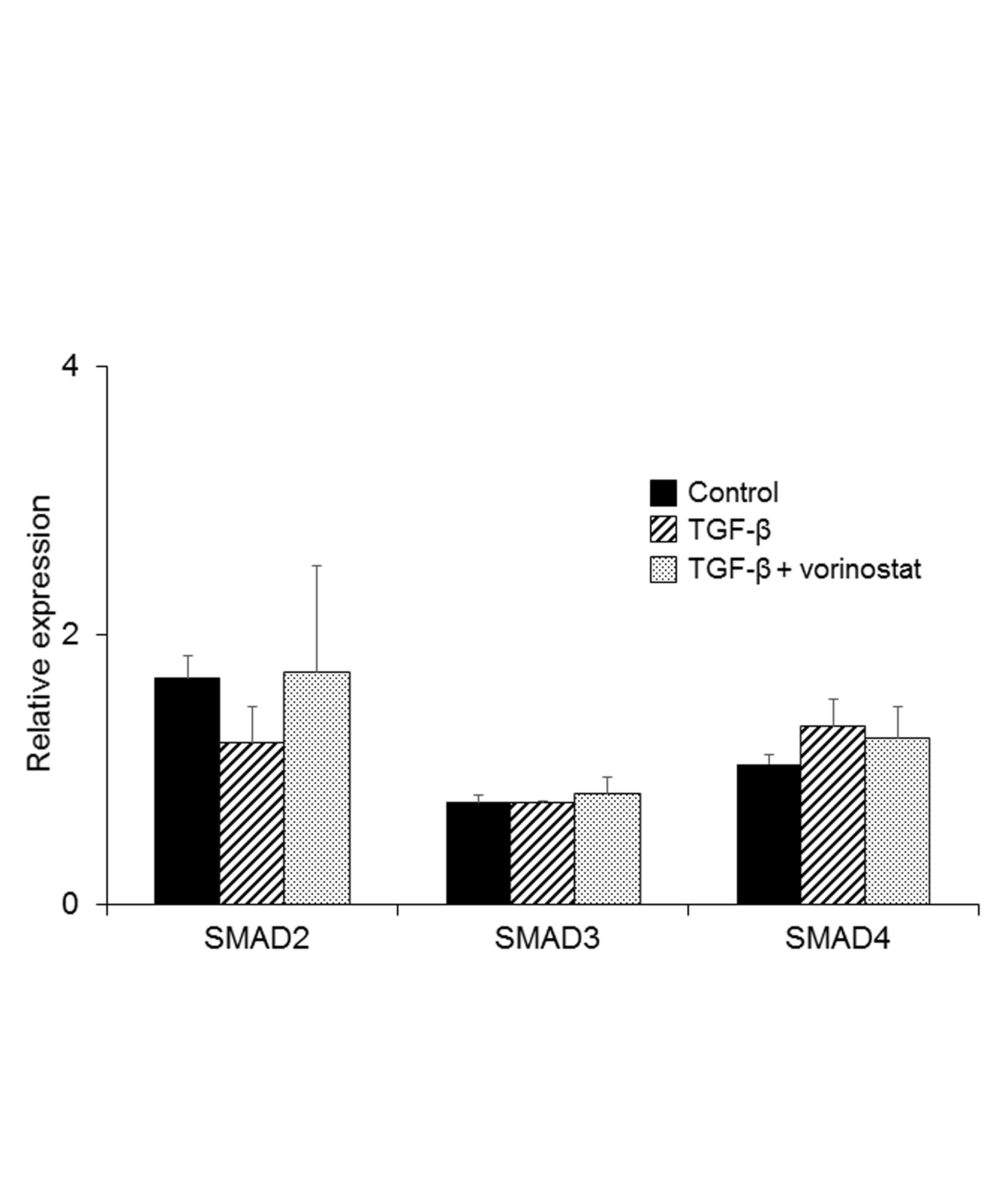

Supplement: S2 Fig — Each cell line was cultured with or without 5 ng/ml of TGF-β1 and 100 nM of vorinostat for 72 h. Then, SMAD2, SMAD3, and SMAD4 were assessed by qRT-PCR. Values represent the mean ± S.D. All experiments were conducted at least three times. (TIF) [file pone.0145985.s002.TIF]

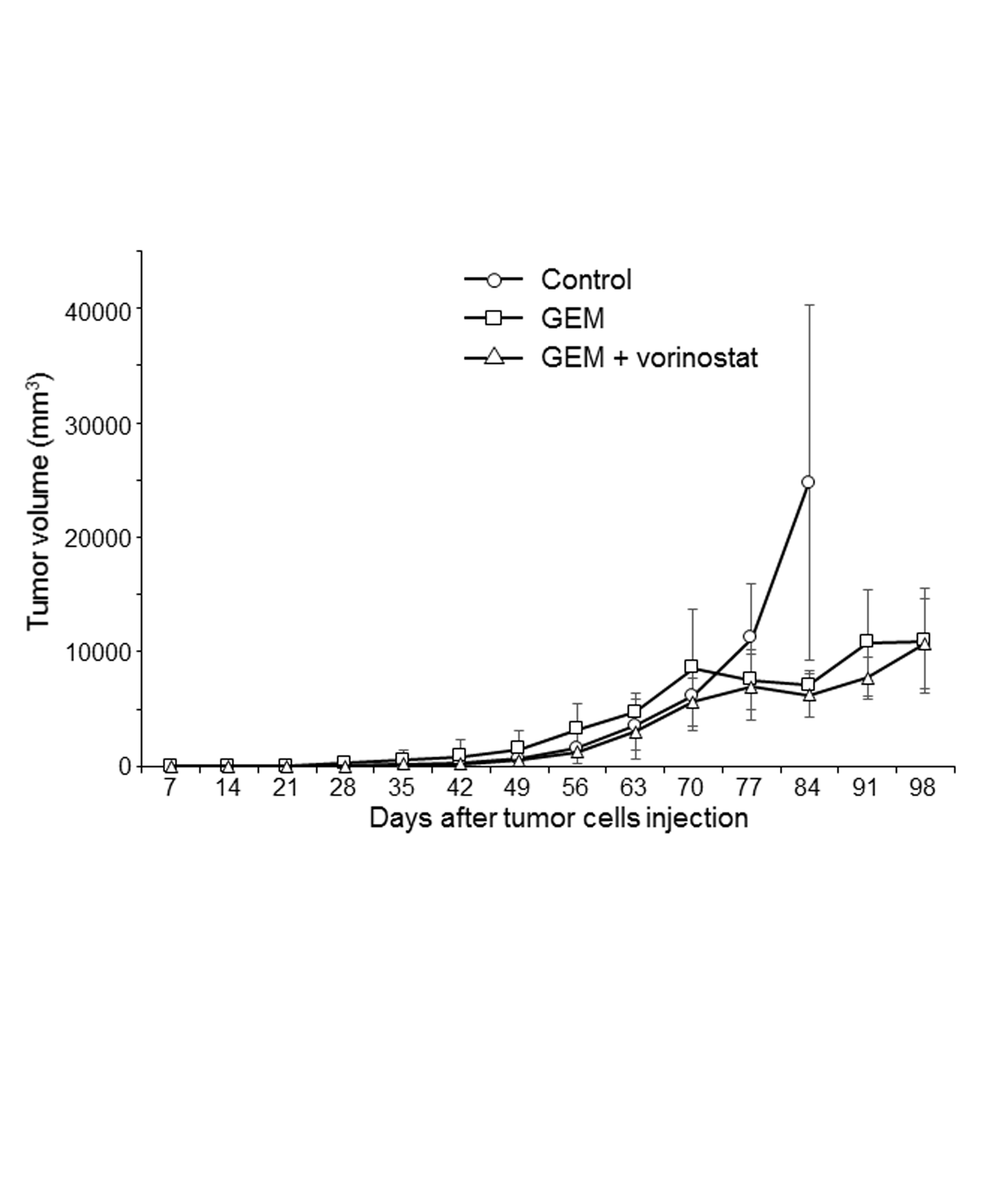

Supplement: S3 Fig — Tumor progression was assessed by tumor volume. Values represent the mean ± S.D. (TIF) [file pone.0145985.s003.TIF]

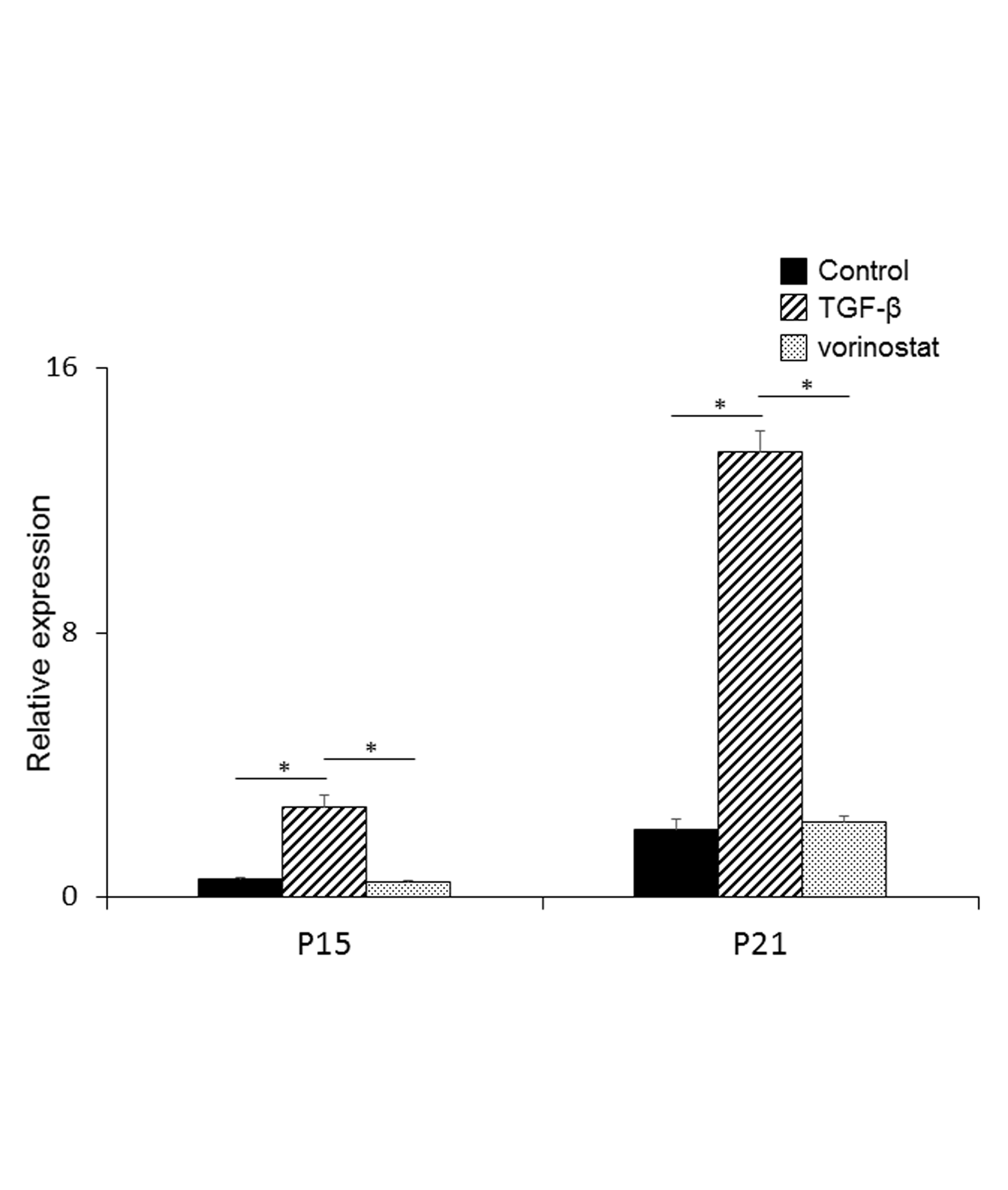

Supplement: S4 Fig — Each cell line was cultured with or without 5 ng/ml of TGF-β1 and 100 nM of vorinostat for 72 h. Then, p15 and p21 were assessed by qRT-PCR. Values represent the mean ± S.D. *P < 0.05. All experiments were conducted at least three times. (TIF) [file pone.0145985.s004.TIF]
