# Supplementary material for: A Histone Deacetylase Inhibitor Suppresses Epithelial-Mesenchymal Transition and Attenuates Chemoresistance in Biliary Tract Cancer
Source: PLoS One. 2016 Jan 4;11(1):e0145985. doi: 10.1371/journal.pone.0145985 (PMC4699768; doi:10.1371/journal.pone.0145985)
Supplement: S1 Table — (DOCX) [file pone.0145985.s005.docx]

S1A Table. Cytokines associated with EMT.

| Cytokines | Relevance to EMT | Reference Number |
| --- | --- | --- |
| TGF-β | Induce EMT via TGFβ/SMAD pathway | #1 |
| BMP | Induce EMT via BMP/SMAD pathway | #2 |
| IL-6 | Induce EMT by interaction with TGFβ | #3 |
| IL-8 | Maintenance of tumor EMT | #4 |
| IL-10 | Promote EMT in pancreatic cancer | #5 |
| TNF-α | Increase the expression of transcription factor Snail | #6 |
| IFN | Control of EMT/MET programs of tumor cells | #7 |
| HGF | Induce EMT with c-Met | #8 |
| IGF | Induce EMT | #9 |
| FGF | Contribute to the mechanisms of EMT by stimulating proteases in the microenvironment | #10 |

Abbreviations: TGF-β, transforming growth factor-beta; BMP, bone morphogenetic protein; IL-4, interleukin-4; IL-6, interleukin-6; IL-17A, interleukin-17A; IL-8, interleukin-8; IL-10, interleukin-10; TNF-α; tumor necrosis factor-alpha; IFN, interferon; HGF, hepatocyte growth factor; IGF, Insulin-like growth factor; FGF, fibroblast growth factor

S1B Table. References for S1A Table.

| Reference Number | Reference (First Author, Year of Publication, Journal) |
| --- | --- |
| #1 | Zavadil J, 2005, Oncogene, 24: 5764-74. |
| #2 | Gonzalez DM, 2014, Sci Signal, 7(344): re8. |
| #3 | Yamada D, 2013, Eur J Cancer, 49: 1725-40. |
| #4 | Fernando RI, 2011, Cancer Res, 71(15): 5296-306. |
| #5 | Liu CY, 2013, Lab Invest, 93(7): 844-54. |
| #6 | Wang H, 2013, Eur J Pharmacol, 714(1-3): 48-55. |
| #7 | Sung JY, 2014, Cell Death Dis, 5: e1224. |
| #8 | Ogunwobi OO, 2013, PLoS One, 8: e63765. |
| #9 | Li H, 2015, Oncol Lett, 9(1): 143-148. |
| #10 | Strutz F, 2002, Kidney Int, 61(5): 1714-28. |
