# Supplementary material for: A Histone Deacetylase Inhibitor Suppresses Epithelial-Mesenchymal Transition and Attenuates Chemoresistance in Biliary Tract Cancer
Source: PLoS One. 2016 Jan 4;11(1):e0145985. doi: 10.1371/journal.pone.0145985 (PMC4699768; doi:10.1371/journal.pone.0145985)
Supplement: S2 Table — (DOCX) [file pone.0145985.s006.docx]

S2 Table. Antibodies used in western blot analysis, immunohistochemistry, and immunocytochemistry.

| Antibody | Source | Dilution | Purchased From |
| --- | --- | --- | --- |
| anti-CDH1 | rabbit polyclonal | 1:200 | Santa Cruz Biotechnology |
| anti-CDH2 | mouse monoclonal | 1:100 | Santa Cruz Biotechnology |
| anti-Smad2/3 | rabbit polyclonal | 1:1000 | Cell Signaling Technology |
| anti-p-Smad2/3 | goat polyclonal | 1:200 | Santa Cruz Biotechnology |
| anti-Smad4 | mouse monoclonal | 1:100 | Santa Cruz Biotechnology |
| anti-JNK | rabbit polyclonal | 1:200 | Santa Cruz Biotechnology |
| anti-p-JNK | mouse monoclonal | 1:200 | Santa Cruz Biotechnology |
| anti-Histone H3 | rabbit polyclonal | 1:1000 | Cell Signaling Technology |
| anti-β-actin antibody | rabbit monoclonal | 1:1000 | Sigma-Aldrich |
