# Supplementary material for: A Histone Deacetylase Inhibitor Suppresses Epithelial-Mesenchymal Transition and Attenuates Chemoresistance in Biliary Tract Cancer
Source: PLoS One. 2016 Jan 4;11(1):e0145985. doi: 10.1371/journal.pone.0145985 (PMC4699768; doi:10.1371/journal.pone.0145985)
Supplement: S3 Table — (DOCX) [file pone.0145985.s007.docx]

S3 Table. Primers used in qRT-PCR.

| Gene-specific primers | Sequences (5′ to 3′) |
| --- | --- |
| HDAC1 forward | CTTCCCCAACCCCTCAGATT |
| HDAC1 reverse | ATCCCTTTCACCCAGACCTG |
| HDAC2 forward | TGGTGTCCAGATGCAAGCTA |
| HDAC2 reverse | GCCACATTTCTTCGACCTCC |
| HDAC3 forward | ACTTCGAGTACTTTGCCCCA |
| HDAC3 reverse | GGCACGTCATGAATCTGGAC |
| HDAC8 forward | ACGTGTCTGATGTTGGCCTA |
| HDAC8 reverse | TCCCAGCTGTAAGACCACTG |
| CDH1 forward | GAGAAACAGGATGGCTGAAGG |
| CDH1 reverse | TGAGGATGGTGTAAGCGATGG |
| CDH2 forward | TGTTTGACTATGAAGGCAGTGG |
| CDH2 reverse | TCAGTCATCACCTCCACCAT |
| VIM forward | AGCTAACCAACGACAAAGCC |
| VIM reverse | TCCACTTTGCGTTCAAGGTC |
| SNAI1 forward | CGAGATCCCTCCAAAATCAA |
| SNAI1 reverse | TTCACACCCATGACGAACAT |
| SMAD2 forward | CGTCCATCTTGCCATTCACG |
| SMAD2 reverse | CTCAAGCTCATCTAATCGTCCTG |
| SMAD3 forward | CCATCTCCTACTACGAGCTGAA |
| SMAD3 reverse | CACTGCTGCATTCCTGTTGAC |
| SMAD4 forward | CCACCAAGTAATCGTGCATCG |
| SMAD4 reverse | TGGTAGCATTAGACTCAGATGGG |
| p15 forward | CTAGTGGAGAAGGTGCACA |
| p15 reverse | TCATCATGACCTGGATCGCG |
| p21 forward | TGGAGACTCTCAGGGTCGAAA |
| p21 reverse | GGCGTTTGGAGTGGTAGAAAT |
| ACTB forward | GCGTTACACCCTTTCTTGAC |
| ACTB reverse | TTGTGAACTTTGGGGGATGC |
