# Supplementary material for: A Histone Deacetylase Inhibitor Suppresses Epithelial-Mesenchymal Transition and Attenuates Chemoresistance in Biliary Tract Cancer
Source: PLoS One. 2016 Jan 4;11(1):e0145985. doi: 10.1371/journal.pone.0145985 (PMC4699768; doi:10.1371/journal.pone.0145985)
Supplement: S4 Table — (DOCX) [file pone.0145985.s008.docx]

S4A Table. Clinical Trials involving HDAC inhibitors.

| Reports | Primary | Patients | Drugs | Other therapies | DCR (%) | Reference Number |
| --- | --- | --- | --- | --- | --- | --- |
| Richards DA (2006) | Pancreatic cancer | 85 | CI-994 | Gemcitabine | 12 | #1 |
| Stadler WM (2006) | Renal cell cancer | 29 | Depsipeptide | Alone | 7 | #2 |
| Sharma S (2008) | Prostate cancer | 10 | VPA | Alone | 20 | #3 |
| Vansteenkiste J (2008) | Lung cancer (NSCLC) | 16 | Vorinostat | Alone | 50 | #4 |
| Woyach JA (2009) | Thyroid carcinoma | 19 | Vorinostat | Alone | 0 | #5 |
| Bradley D (2009) | Prostate | 27 | Vorinostat | Alone | Failure | #6 |
| Anne M (2009) | Lung cancer (NSCLC) | 14 | Vorinostat | Carboplatin | - | - |
| Wilson PM (2010) | Colorectal cancer | 10 | Vorinostat | 5-FU | Failure | #7 |
| Suresh S (2010) | Lung cancer (NSCLC) | 94 | Vorinostat | Carboplatin  + paclitaxel | - | - |
| Molife LR (2010) | Prostate cancer | 35 | Romidepsin | Alone | - | - |
| Munster PN (2011) | Breast cancer | 43 | Vorinostat | Tamoxifen | 40 | #8 |
| Hainsworth JD (2011) | Renal cell cancer | 20 | Panobinostat | Alone | 0 | #9 |
| Dizon DS (2012) | Gynecologic oncology | 35 | Belinostat | Alone | 63 | #10 |
| Ramaswany B (2012) | Breast cancer | 44 | Vorinostat | Paclitaxel | 55 | #11 |
| Fakih MG (2012) | Colorectal cancer | 58 | Vorinostat | 5-FU | 53 | #12 |
| Tu Y (2014) | Breast cancer | 55 | Vorinostat | Paclitaxel | 33 | #13 |
| Krug LM (2015) | Pleural mesothelioma | 329 | Vorinostat | Alone | - | #14 |

Abbreviations: HDAC, histone deacetylase; DCR, disease control rate; NSCLC, non-small cell lung cancer; HCC, hepatocellular carcinoma, 5-FU, 5-Fluorouracil

S4B Table. References for S4A Table

| Reference Number | Reference (First Author, Year of Publication, Journal) |
| --- | --- |
| #1 | Richards DA, 2006, Ann Oncol, 17(7): 1096-102. |
| #2 | Stadler WM, 2006, Clin Genitourin Cancer, 5(1): 57-60. |
| #3 | Sharma S, 2008, Transl Oncol, 1(3): 141-7. |
| #4 | Vansteenkiste J, 2008, Invest New Drugs, 26(5): 483-8. |
| #5 | Woyach JA, 2009, J Clin Endocrinol Metab, 94(1): 164-70. |
| #6 | Bradley D, 2009, Cancer, 115(23): 5541-9. |
| #7 | Wilson PM, 2010, Cancer Chemother Pharmacol, 65(5): 979-88. |
| #8 | Munster PN, 2011, Br J Cancer, 104(12): 1828-35. |
| #9 | Hainsworth JD, 2011, Cancer Invest, 29(7): 451-5. |
| #10 | Dizon DS, 2012, Int J Gynecol Cancer, 22(6): 979-86. |
| #11 | Ramaswamy B, 2012, Breast Cancer Res Treat, 132(3): 1063-72. |
| #12 | Fakih MG, 2012, Cancer Chemother Pharmacol, 69(3): 743-51. |
| #13 | Tu Y, 2014, Breast Cancer Res Treat, 146(1): 145-52. |
| #14 | Krug LM, 2015, Lance Oncol, 16(4): 447-56. |
